# Construction of Genetically Encoded Biosensors to Monitor Subcellular Compartment-Specific Glutathione Response to Chemotherapeutic Drugs in Acute Myeloid Leukemia Cells

**DOI:** 10.1021/acs.analchem.2c04255

**Published:** 2023-01-26

**Authors:** Ghulam Abbas, Mengmeng Cui, Dianbing Wang, Min Li, Xian-En Zhang

**Affiliations:** † National Laboratory of Biomacromolecules, Institute of Biophysics, Chinese Academy of Sciences, Beijing 100101, China; ‡ Faculty of Synthetic Biology, Shenzhen Institute of Advanced Technology, Chinese Academy of Sciences, Shenzhen 518055, China; § University of Chinese Academy of Sciences, Beijing 100049, China

## Abstract

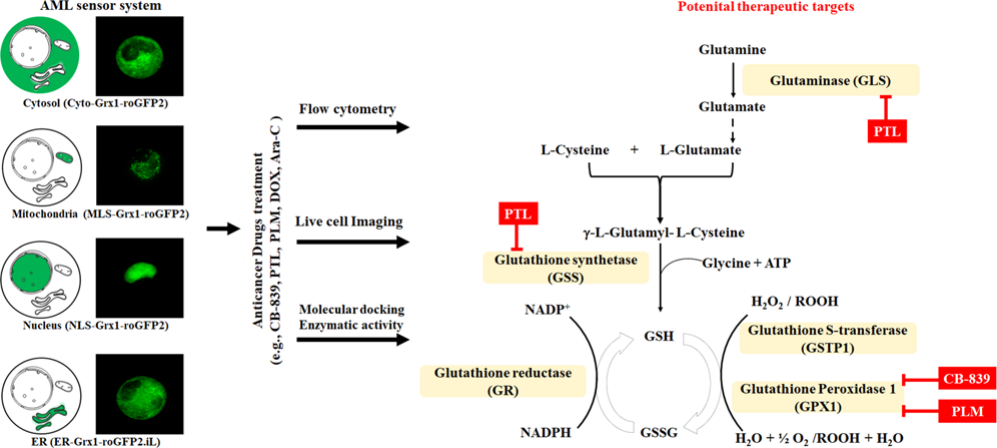

Glutathione (GSH), the constituent of the redox buffer system, is a scavenger of reactive oxygen species (ROS), and its ratio to oxidized glutathione (GSSG) is a key indicator of oxidative stress in the cell. Acute myeloid leukemia (AML) is a highly aggressive hematopoietic malignancy characterized by aberrant levels of reduced and oxidized GSH due to oxidative stress. Therefore, the real-time, dynamic, and highly sensitive detection of GSH/GSSG in AML cells is of great interest for the clinical diagnosis and treatment of leukemia. The application of genetically encoded sensors to monitor GSH/GSSG levels in AML cells is not explored, and the underlying mechanism of how the drugs affect GSH/GSSG dynamics remains unclear. In this study, we developed subcellular compartment-specific sensors to monitor GSH/GSSG combined with high-resolution fluorescence microscopy that provides insights into basal GSH/GSSG levels in the cytosol, mitochondria, nucleus, and endoplasmic reticulum of AML cells, in a decreasing order, revealing substantial heterogeneity of GSH/GSSG level dynamics in different subcellular compartments. Further, we investigated the response of GSH/GSSG ratio in AML cells caused by Prussian blue and Fe_3_O_4_ nanoparticles, separately and in combination with cytarabine, pointing to steep gradients. Moreover, cytarabine and doxorubicin downregulated the GSH/GSSG levels in different subcellular compartments. Similarly, live-cell imaging showed a compartment-specific decrease in response to various drugs, such as CB-839, parthenolide (PTL), and piperlongumine (PLM). The enzymatic activity assay revealed the mechanism underlying fluctuations in GSH/GSSG levels in different subcellular compartments mediated by these drugs in the GSH metabolic pathway, suggesting some potential therapeutic targets in AML cells.

Acute myeloid leukemia (AML) is a very aggressive malignancy of immature myeloblasts that is more common in adults and has a poor prognosis with a high relapse rate.^1,2^ The advancements in different strategies to eliminate AML cells remain a major challenge in combating leukemia. AML cells show aberrant levels of reduced as well as oxidized glutathione and perturbed expression levels of glutathione metabolism and homeostasis-linked enzymes.^3,4^ Glutathione, a ubiquitous tripeptide thiol, is widely recognized as a reactive oxygen species (ROS) scavenger and a fundamental component of the antioxidant system.^5^ The reduced glutathione (GSH) is converted to an oxidized moiety (GSSG) during oxidative stress to maintain redox homeostasis.^6^ It modulates various cellular processes, such as cell signaling pathways, metabolism, defense, proliferation, stress response, and detoxification of oxidants.^7^ The significance of the GSH/GSSG ratio in tumor growth, progression, and drug resistance has been highlighted.^8^ In different types of cancers, GSH-mediated oxidative stress has been reported as a plausible therapeutic target for most drugs. For example, in AML, there is an increased expression of numerous glutathione pathway regulatory proteins to combat oxidative stress. In addition, leukemic cells exhibit a decline in reduced GSH and an increase in oxidized glutathione (GSSG) levels, indicating that AML cells are hypersensitive to chemotherapeutic drug-mediated inhibition of GSH metabolism.^4^ Moreover, the mechanism underlying the development of cancer cell resistance to therapeutic drugs is based on the overexpression of glutamate–cysteine ligase (GCL, GCLC) and glutathione synthetase (GSS) involved in GSH synthesis, leading to an increase in GSH/GSSG.^8^ Thus, it is important to measure the intracellular GSH/GSSG ratio in AML. GSH is found in various subcellular compartments, such as the mitochondria, cytosol, nucleus, and endoplasmic reticulum (ER), and its concentration varies significantly in different organelles.^9−11^ The monitoring of GSH content at the subcellular level reveals the redox state and the pathophysiology of cells, as the ratio of reduced and oxidized forms of glutathione (GSH/GSSG) indicates cellular oxidative stress in many malignancies. The abnormal GSH/GSSG level during oxidative stress in different subcellular compartments is evidence of distinct cellular activity. For example, in leukemic cells, the significant increase in mitochondrial mass is due to the reduction of hydrogen peroxide by glutathione peroxidase (GPX) and the detoxification of electrophiles catalyzed by glutathione S-transferase (GSTP1) to maintain homeostasis.^9,12^ Thus, it is crucial to measure the real-time in vivo and compartment-specific GSH/GSSG-mediated redox differences and fluctuations to comprehend the redox biology and pathophysiology of leukemia cells. In most studies, the GSH/GSSG ratio is determined using chemical and disruptive methods at the whole cell or subcellular level using chemically synthesized trackers in AML cell lysates that disrupt cell integrity, which requires analytical separation methods, such as HPLC, LC/MS, and fluorimetry.^13−16^ However, the application of genetically encoded GSH sensors to overcome these limitations has barely been explored.^17,18^ Due to the “hard-to-transfect” nature of AML cells for exogenous genes,^19,20^ the introduction of the genetically encoded biosensor into AML cells was not accomplished until recently by our group.^21^ Here, we exploited the application of endogenous genetically encoded biosensors comprising human glutaredoxin-1 (Grx1) linked to the redox-sensitive green fluorescent protein, Grx1-roGFP2 (while Grx1-roGFP2.iL for a highly oxidized environment, i.e., ER), fused with subcellular localization guide peptides mitochondria localization sequence (MLS), nuclear localization sequence (NLS), and endoplasmic reticulum localization sequence (ELS) with unprecedented sensitivity and temporal resolution to determine real-time and in vivo GSH/GSSG at the subcellular level.^22−24^ We successfully constructed a sensor system to monitor the basal level of glutathione in four subcellular compartments of AML: the cytosol, nucleus, mitochondria, and ER. Our study demonstrates the utility of genetically encoded redox biosensors in dissecting redox mechanisms, as well as the potential targets determined via enzymatic activity assays associated with the effect of chemotherapeutic drugs on GSH/GSSG levels in different compartments of AML cells, which can present a compelling strategy for better and selective targeting of leukemic cells.

## Experimental Section

### Generation of Stable Cell Lines Using Lentivirus

The HL60 stable cell lines expressing sensor proteins (Cyto-Grx1-roGFP2, MLS-Grx1-roGFP2, NLS-Grx1-roGFP2, ELS-Grx1-roGFP2.iL) were obtained via lentivirus infection and subsequent selection with 3 μg/mL puromycin (Solarbio, Beijing, China). The 293T cells grown at a confluency of 50–60% were cotransfected with three lentiviral packaging vectors (pLPI, pLPII, and pLPVSVG) accompanied by pLVX-sensor vectors (Cyto-Grx1-roGFP2, MLS-Grx1-roGFP2, NLS-Grx1-roGFP2, and ELS-Grx1-roGFP2.iL) using Lipofectamine 3000 (Invitrogen), according to the manufacturer’s protocol, and after 72 h, the supernatant containing the recombinant lentivirus was harvested. We seeded HL60 cells into six-well culture plates for lentiviral infection along with 4 μg/mL Polybrene (Macgene, Beijing, China) and then centrifuged them at 1000*g* for 1 h at 37 °C. Subsequently, the cells were cultured and maintained in a culture medium containing 3 μg/mL puromycin for 1 week, and cells expressing Cyto-Grx1-roGFP2, MLS-Grx1-roGFP2, NLS-Grx1-roGFP2, and ELS-Grx1-roGFP2.iL in the cytosol, mitochondria, nucleus, and endoplasmic reticulum, respectively, were sorted via fluorescence-activated cell sorting (FACS; FACSAria IIIu, BD Biosciences, Franklin Lakes, NJ).

### Treatment of AML Cells with Chemotherapeutic Drugs

DOX and Ara-C were dissolved in absolute ethanol and DMSO, respectively. The HL60 cells were seeded (2 × 10^5^ cells/mL) and treated respectively at 50% confluency with DOX (0–10 μg/mL), Ara-C (0–10 μM), Fe_3_O_4_ NPs (0–100 μg/mL), and PBNPs (0–100 μg/mL) for 24 h. In addition, cells were incubated with a combination of NPs and chemotherapeutic drugs as follows: Fe_3_O_4_ NPs and PBNPs together with 1 μM Ara-C for 24 h, respectively. The final concentration of DMSO and absolute ethanol in the cells was <1%. The controls were always treated with the same amount of solvent.

### Live-Cell Imaging Response of GSH/GSSG Levels of Various Subcellular Compartments of AML Cells to Chemotherapeutic Drugs

Live-cell imaging experiments after treatment with multiple drugs, such as NMM, CB-839, PTL, and PLM, were performed using a Zeiss LSM 980 with a 63× objective lens Airyscan 2 (Zeiss Axio Observer) and a motorized stage to capture multiple view fields controlled by ZEN software. Live-cell imaging was carried out in a temperature-, humidity-, and CO_2_-controlled and regulated chamber. Data were analyzed using ZEN microscopy software. For the live-cell imaging of HL60 suspension cells, the stable cell lines were seeded on a 35 mm glass-bottom dish (BD Biosciences, Franklin Lakes, NJ). The AML cells expressing the probes Cyto-Grx1-roGFP2, MLS-Grx1-roGFP2, and NLS-Grx1-roGFP2 in the cytosol, mitochondria, and nucleus, respectively, were imaged every 5 min for a total of 90 min time series after the addition of a single bolus of 1 mM NMM, 1 μM CB-839, 20 μM PTL, and 20 μM PLM, respectively, carefully to avoid the disturbance and movement of the cells. We acquired the images for the Grx1-roGFP2 at 405 and 488 nm excitation wavelengths and the 525 and 530 nm emission wavelengths. The collected imaging data were processed using Imaris software. All materials, reagents, instruments, and other experimental methods used in this study are described in detail in the Supporting Information.

## Results and Discussion

### Directed Subcellular Localization of GSH/GSSG Probes in AML Cell Stable Lines

**Figure 1:**
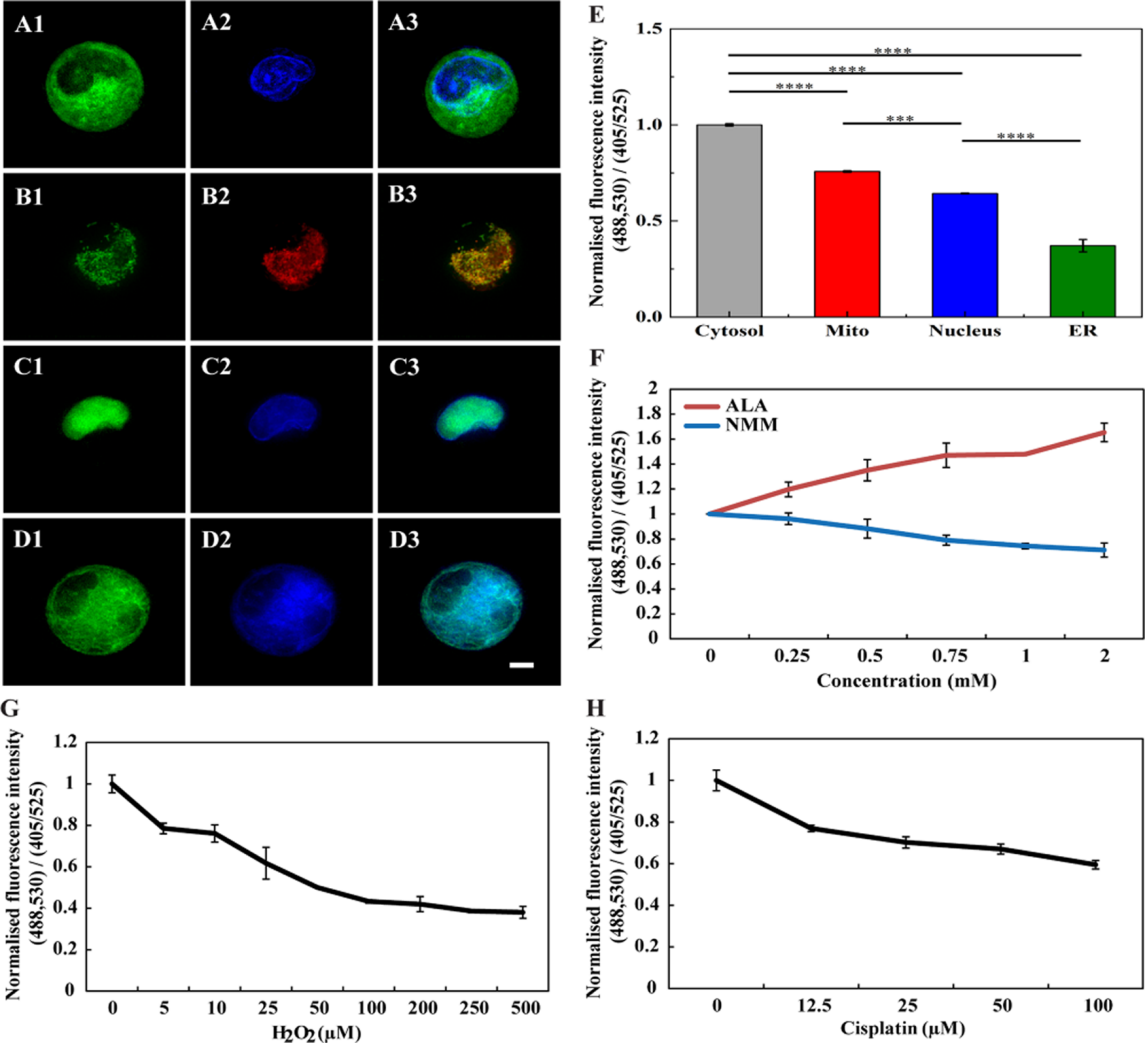
Intracellular localization of Grx1-roGFP2 and Grx1-roGFP2.iL targeted at different subcellular compartments in HL60 cells and the basal GSH/GSSG levels as well as characterization of the GSH/GSSG sensor by measuring the changes in the fluorescence signal intensity of Cyto-Grx1-roGFP2 stably expressed in HL60 cells in response to ALA, NMM, H_2_O_2_, and cisplatin. Panels represent the live-cell imaging of Grx1-roGFP2/ELS-Grx1-roGFP2.iL stably expressed in the cytosol (A1, A2, and A3), mitochondria (B1, B2, and B3), nucleus (C1, C2, and C3), and endoplasmic reticulum (ER) (D1, D2, and D3) of HL60 cells using SIM. Panels A2 and C2 show Hoechst 33342 staining, B2 shows Mito-Tracker Red, and D2 shows ELS-Tracker Blue, whereas panels A3–D3 are merged images. Panel E shows the basal level of glutathione in different subcellular compartments measured using the Grx1-roGFP2/ELS-Grx1-roGFP2.iL. The normalized fluorescence intensity response ratio (488/405 nm) of Cyto-Grx1-roGFP2 stably expressed in the cytosol of HL60 cells (F) 30 min after ALA (0–2 mM) and 60 min after NMM (0–2 mM), (G) 2 min after H_2_O_2_ (0–500 μM), and (H) 24 h after cisplatin (0–100 μM) treatment measured using flow cytometry (≥10,000 cells), whereas 488 and 405 nm as excitation wavelengths, as well as 530/30 and 525/50 nm filter sets for emission, were used respectively. Data are presented as the mean ± SD, *n* ≥ 3, while the statistically significant differences between different groups (****P < 0.0001, ***P < 0.001, **P < 0.01, and *P < 0.05) were calculated via the one-way analysis of variance (ANOVA) using GraphPad software.

The in vivo GSH/GSSG levels in various subcellular compartments have been measured using genetically encoded biosensors or chemical probes in several types of human cell lines like HeLa, 293T, Jurkat, and A549 cell.^25,26^ In particular, the Grx1-roGFP2 and Grx1-roGFP2.iL can have a specific fluorescent response to the GSH/GSSG ratio when excited with 408/488 nm laser.^22,23^ However, as mentioned previously, due to the “hard-to-transfect” nature^19,20^ of AML cells, exogenous gene expression has barely been explored in AML cells. In the current study, the genetically encoded fluorescent probes, Grx1-roGFP2/Grx1-roGFP2.iL, were employed to design and construct an AML sensor system based on lentiviral transduction to investigate GSH/GSSG fluctuations within various subcellular compartments such as cytosol, nucleus, mitochondria, and ER. The recombinant lentivirus was used to infect the HL60 type of AML cells to generate four stable cell lines expressing GSH-specific fluorescent indicators in the cytosol, nucleus, mitochondria, or ER. Live-cell imaging confirmed the exact localization and expression of Grx1-roGFP2/Grx1-roGFP2.iL in different subcellular compartments (Figure 1). The cells expressing Cyto-Grx1-roGFP2 exclusively exhibited a uniform cytosolic distribution of fluorescent protein sensor signals compared to the Hoechst 33342-stained nucleus (Figure 1A1–A3). MLS-Grx1-roGFP2 was intended to target the mitochondria and colocalize with Mito-Tracker Red (Figure 1B1–B3), and the PCC between the MLS-Grx1-roGFP2 probe and Mito-Tracker signal was recorded as 0.9507 (Figure S1A), indicating the correct distribution of MLS-Grx1-roGFP2 in the mitochondria. The nuclear localization of NLS-Grx1-roGFP2 was also verified through colocalization with Hoechst 33342 (Figure 1C1–C3), and the PCC between the NLS-Grx1-roGFP2 and Hoechst signal was calculated as 0.9352 (Figure S1B), which exhibits the successful nuclear localization of NLS-Grx1-roGFP2. The homogeneous colocalization of ELS-Grx1-roGFP2.iL and ELS-Tracker Red (Figure 1D1–D3) showed a PCC value of 0.9705 (Figure S1C), confirming the accurate ELS-Grx1-roGFP2.iL expression in the ER. Eventually, the live-cell imaging data confirmed the successful construction of four stable AML cell lines expressing fluorescent glutathione probes in the cytosol, mitochondria, nucleus, and ER, respectively.

### Basal GSH/GSSG Levels in Four Subcellular Compartments in AML Cells

To evaluate the mechanism behind the transportation and the communication of GSH/GSSH among different compartments in the AML cells during the disease onset, the subcellular basal GSH/GSSG level was investigated using the sensor system AML cells. The basal levels of GSH/GSSG were measured in the cytosol, mitochondria, nucleus, and ER of AML cells using Cyto-Grx1-roGFP2, MLS-Grx1-roGFP2, NLS-Grx1-roGFP2, and ELS-Grx1-roGFP2.iL, respectively. The results provided evidence of variability in GSH/GSSG levels in different subcellular compartments (Figure 1E), as they were the highest in the cytosol, followed by the mitochondria and nucleus. The ER, having a highly oxidized environment,^27^ exhibited the lowest GSH/GSSG levels determined using ELS-localized Grx1-roGFP2.iL (Figure 1E). Our findings in AML cells demonstrated a similar trend as described in other mammalian cells in previous studies,^27−30^ except for the nuclear basal level, which was still unclear or identical to the cytosolic level. Besides, the basal levels of GSH/GSSG in these three compartments were significantly different from each other. It was reported that the reduced and mtGSH pools are restored by NADPH-dependent mitochondrial oxidoreductases,^31^ while transporters or channels maintain the mitochondrial glutathione pool to allow GSH to cross the mitochondrial membrane barrier and diffuse from the cytosol.^32^ Similarly, the nucleus and ER lack specific enzymes required for GSH biosynthesis, conferring lower GSH levels. Therefore, GSH is usually replenished and transported to all of the compartments via specific channels and pores.^30,32−34^ The compartment-specific GSH basal level could give insights into the homeostasis and redox condition of the AML cells and could lead to determining the pathophysiological state of the AML patients.

### Characterization of the GSH/GSSG Sensor in AML Stable Cell Lines

The ratiometric calibration was performed in the Cyto-Grx1-roGFP2-expressing stable AML cell line using flow cytometry to characterize the sensitivity and specificity of Grx1-roGFP2 to GSH/GSSG levels. The HL60 cells were also treated with H_2_O_2_ as it was used for in vitro characterization of Grx1-roGFP2 by monitoring the changes in GSH/GSSG levels of AML cells as regulated by H_2_O_2_. It was observed that H_2_O_2_ a typical oxidant can directly decrease the intracellular GSH/GSSG levels. Notably, the H_2_O_2_ could not induce a substantial sensor response signal in stable cell lines. Instead, it was due to the H_2_O_2_-mediated intracellular GSH regulation ensuring that GSH caused and preceded the sensor response signal.^22^ Besides, the stable cell lines were treated with the typical GSH enhancer ALA and inhibitor NMM. The normalized fluorescence intensity response ratio (488/405 nm) was recorded for Cyto-Grx1-roGFP2 stably expressed in the cytosol of HL60 cells 30 min after ALA (0–2 mM) and 60 min after NMM (0–2 mM) (Figure 1F), 2 min after H_2_O_2_ (0–500 μM) (Figure 1G), and 24 h after cisplatin (0–100 μM) (Figure 1H) treatment via flow cytometry (≥10,000 cells), whereas 488 and 405 nm used as excitation wavelengths as well as 530/30 and 525/50 nm filter sets for emission, respectively. Cyto-Grx1-roGFP2 was responsive to ALA, NMM, H_2_O_2_, and cisplatin, respectively, in a concentration-dependent manner and showed a ratiometric increase in the fluorescence signal upon dual excitation at 488 and 405 nm. Additionally, the live-cell imaging of stable cell lines expressing GSH/GSSG sensors in different subcellular compartments also showed a decrease in fluorescence signal intensity in response to NMM (1 mM)-treated AML cells in the (Figure S2A) cytosol, (Figure S2C) mitochondria, and (Figure S2E) nucleus. The quantification analysis of live-cell imaging exhibited that the normalized fluorescence signal intensity ratio (488,530/405,525 nm) of probes Cyto-Grx1-roGFP2 (Figure S2B), MLS-Grx1-roGFP2 (Figure S2D), and NLS-Grx1-roGFP2 (Figure S2F) decreased as plotted over time in response to NMM, whereas Figure S2G represents the viability of NMM-treated HL60 cells after 24 h. It is known that the GSH enhancer (ALA) increased the GSH levels in A549 cells, whereas the inhibitor (NMM) caused a decrease in GSH levels.^26^ Likewise, in the current study, the sensor in AML cells responded to the fluctuations in the GSH/GSSG levels regulated by ALA and NMM. These results showed that the sensors are functional in AML cells which agree with studies carried out in other mammalian cells.^22,23,35^

### Changes in GSH/GSSG Levels in Various Subcellular Compartments of AML Cells in Response to Chemotherapeutic Drugs

**Figure 2:**
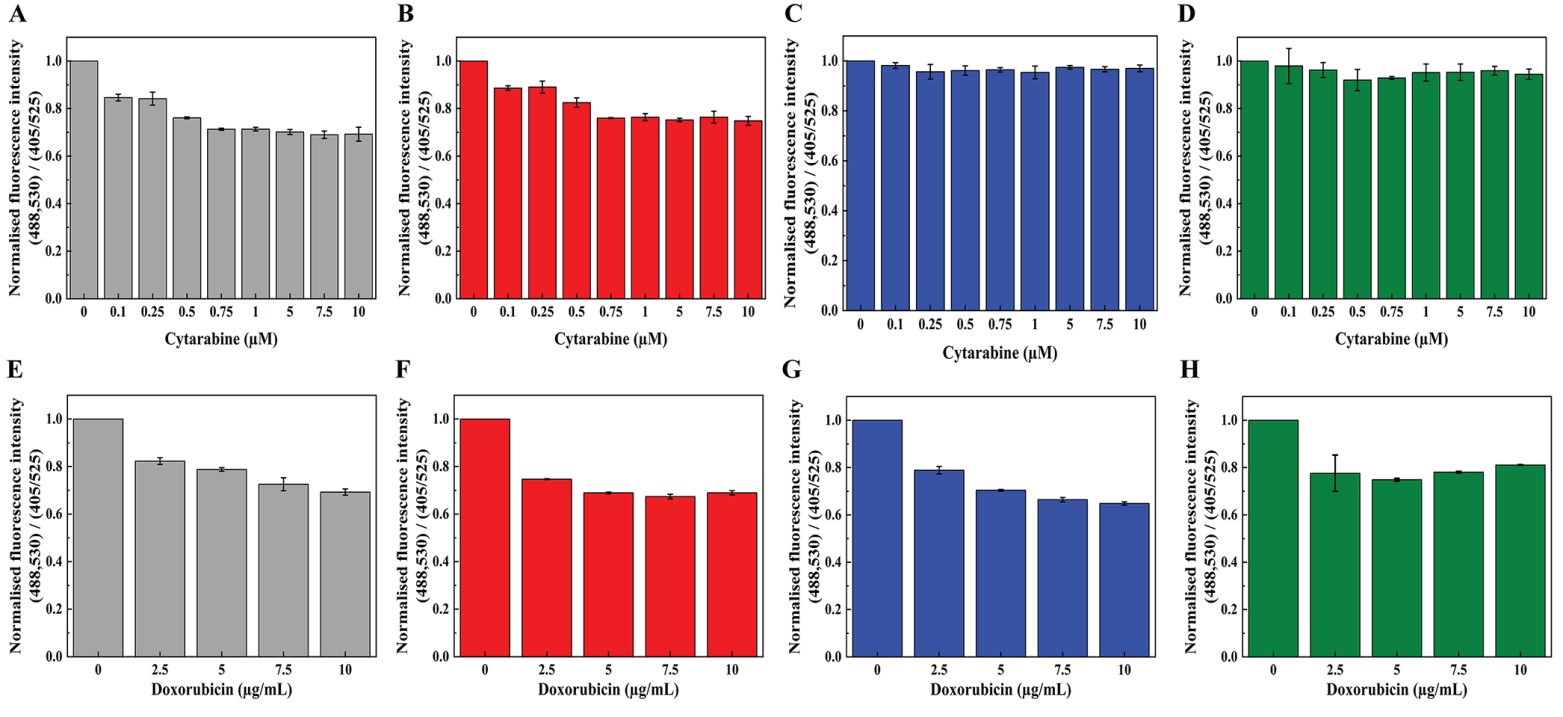
Change in GSH/GSSG levels in various subcellular compartments in response to antitumor drugs in HL60 cells stably expressing Grx1-roGFP2/Grx1-roGFP2.iL. The normalized fluorescence signal response ratio (488/405 nm) of Cyto-Grx1-roGFP2, MLS-Grx1-roGFP2, NLS-Grx1-roGFP2, and ELS-Grx1-roGFP2.iL to cytarabine in the (A) cytosol, (B) mitochondria, (C) nucleus, and (D) ER and that to DOX in the (E) cytosol, (F) mitochondria (G) nucleus, and (H) ER, respectively, after 24 h of treatment determined using flow cytometry (≥10,000 cells). Bandpass 530/30 and 525/50 nm emission filters were used for 488 and 405 nm excitation wavelengths, respectively. One-way ANOVA was used to compare the differences between different groups. Data are presented as the mean ± SD, *n* ≥ 3.

To evaluate the subcellular GSH/GSSG levels in specific subcellular compartments of AML cells in response to common chemotherapeutic drugs, four AML stable cell lines (Cyto-Grx1-roGFP2, MLS-Grx1-roGFP2, NLS-Grx1-roGFP2, and ELS-Grx1-roGFP2.iL) were, respectively, treated with cytarabine (Ara-C) and DOX at different concentrations for 24 h. Previously, the total GSH/GSSG levels were determined in human AML cells in response to Ara-C using DTNB colorimetric methods.^4^ Here, the response ratio demonstrated that Cyto-Grx1-roGFP2 and MLS-Grx1-roGFP2 were responsive to cytarabine ranging from 0 to 10 μM in a concentration-dependent manner, revealing that cytarabine decreased the GSH/GSSG levels in the cytosol and mitochondria in accordance to the correlation between GSH/GSSG levels and the fluorescence signal ratio of Grx1-roGFP2 (Figure 2A,B), conferring to be the most sensitive compartments to the anticancer drug. However, the NLS-Grx1-roGFP2 and ELS-Grx1-roGFP2.iL exhibited minute changes in the GSH/GSSG level of the nucleus and the ER in response to cytarabine as compared to the cytosol and mitochondria (Figure 2C,D). The response ratio of the sensor suggested that all Cyto-Grx1-roGFP2, MLS-Grx1-roGFP2, and NLS-Grx1-roGFP2 were responsive to DOX ranging from 0 to 10 μM in a dose-dependent manner, indicating that DOX depleted the GSH/GSSG levels in the cytosol, mitochondria, and nucleus.

At the same time, in the ER, it was increased slightly after the concentration was maximum. Still, the same depletion pattern of GSH/GSSG levels was observed in the ER as compared to the control group, revealing that the cytosol, mitochondria, and nucleus were the most sensitive subcellular compartments while the ER was comparably less sensitive to the DOX (Figure 2E–H). While the DOX was reported to decrease GSH/GSSG levels in target tissues and cancers such as human colon carcinoma cells (Caco-2), as well as human hepatocellular carcinoma cells (HepG2) but the specific subcellular level remained unexplored.^36,37^ Notably, by measuring the basal GSH/GSSG level of different subcellular compartments, we are able to investigate the effects of chemotherapeutic drugs on GSH/GSSG levels at the subcellular level in AML cells, which would help to elucidate the drug’s mechanism of action in AML therapy. Furthermore, the sensor also responded to the changes caused by the nanoparticles (PBNPs and Fe_3_O_4_ NPs (Figure S3)) to the GSH/GSSG level in AML cells. As reported previously, the Fe_3_O_4_ NPs induced the depletion of GSH and increased the ROS level in different cancer cells, e.g., HepG2 and A549 selectively kill cancer cells via the P53 pathway^42,43^ and PBNPs acted as multienzyme mimetics and ROS scavengers to protect the cells against oxidative stress, which could be the reason for the increase in GSH/GSSG levels.^44^

### Live-Cell Imaging of the Changes in GSH/GSSG Levels in Various Subcellular Compartments of AML Cells in Response to Chemotherapeutic Drugs

**Figure 3:**
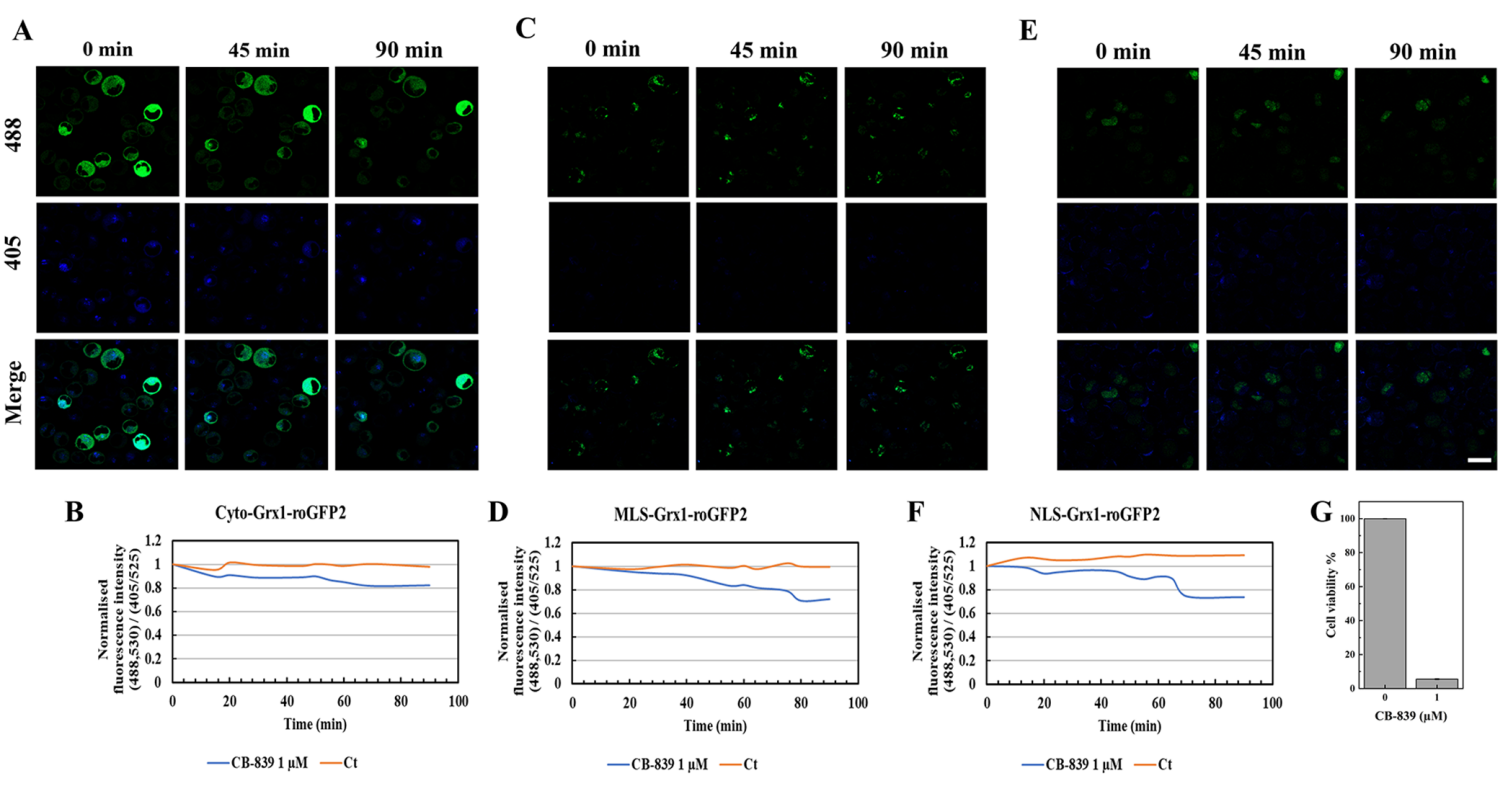
Representative live-cell images of the changes in GSH/GSSG levels in various subcellular compartments (cytosol, mitochondria, and nucleus) of AML cells in response to the glutaminase inhibitor CB-839 (1 μM). The AML cells expressing the probes Cyto-Grx1-roGFP2, MLS-Grx1-roGFP2, and NLS-Grx1-roGFP2 in the (A) cytosol, (C) mitochondria, and (E) nucleus, respectively, imaged every 5 min for a total of 90 min series after treatment with 1 μM CB-839. Quantification of live-cell imaging results showing the normalized fluorescence signal intensity ratio (488,530/405,525) of (B) Cyto-Grx1-roGFP2, (D) MLS-Grx1-roGFP2, and (F) NLS-Grx1-roGFP2 measured using Imaris software and plotted over time in response to CB-839. Scale bar: 20 μm. (G) Cell viability of CB-839-treated HL60 cells after 24 h, whereas the treatment group is significantly different from the control group. One-way ANOVA was used to compare the differences between different groups. Data are presented as the mean ± SD, *n* ≥ 3.

The GSH/GSSG level-targeting efficiency of various chemotherapeutic drugs in different subcellular compartments of AML suspension cells was evaluated using live-cell imaging. The drugs CB-839, PTL, and PLM were used to treat the AML stable cell lines to target the GSH/GSSG levels, as observed via live-cell imaging. The imaging data suggested that the treatment of AML stable cell lines expressing the probes Cyto-Grx1-roGFP2 (Figure 3A), MLS-Grx1-roGFP2 (Figure 3C), and NLS-Grx1-roGFP2 (Figure 3E) with 1 μM CB-839 resulted in the decrease and depletion of GSH/GSSG levels in different subcellular compartments, including the cytosol, mitochondria, and nucleus, but the mitochondria and nucleus were found to be the most sensitive compartments. The fluorescence signal intensity of different subcellular compartments such as the cytosol (Figure 3B), mitochondria (Figure 3D), and nucleus (Figure 3F) also substantiate the results obtained using live-cell imaging. Moreover, the CCK8 assay suggested that the viability of HL60-type AML cells was significantly reduced upon exposure to 1 μM CB-839 for 24 h (Figure 3G).

**Figure 4:**
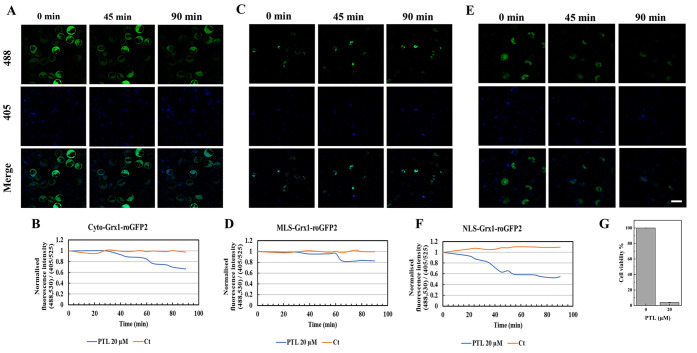
Representative live-cell images of the changes in GSH/GSSG levels in various subcellular compartments (cytosol, mitochondria, and nucleus) of AML cells in response to the glutaminase inhibitor PTL (20 μM). The AML cells expressing the probes Cyto-Grx1-roGFP2, MLS-Grx1-roGFP2, and NLS-Grx1-roGFP2 in the (A) cytosol, (C) mitochondria, and (E) nucleus, respectively, imaged every 5 min for a total of 90 min series after treatment with 20 μM PTL. Quantification of live-cell imaging results showing the normalized fluorescence signal intensity ratio (488,530/405,525) of (B) Cyto-Grx1-roGFP2, (D) MLS-Grx1-roGFP2, and (F) NLS-Grx1-roGFP2 measured using Imaris software and plotted over time in response to PTL. Scale bar: 20 μm. (G) Cell viability of PTL-treated HL60 cells after 24 h, whereas the treatment group is significantly different from the control group. One-way ANOVA was used to compare the differences between different groups. Data are presented as the mean ± SD, *n* ≥ 3.

Similarly, the treatment of AML stable cell lines expressing different subcellular targeted probes, Cyto-Grx1-roGFP2 (Figure 4A), MLS-Grx1-roGFP2 (Figure 4C), and NLS-Grx1-roGFP2 (Figure 4E), with 20 μM PTL caused a decline in the GSH/GSSG levels in different subcellular compartments, including cytosol, mitochondria, and nucleus, but primarily in the cytosol and nucleus, which were identified as the most sensitive compartments for GSH/GSSG level perturbations. The fluorescence signal intensity in distinct subcellular compartments such as the cytosol (Figure 4B), mitochondria (Figure 4D), and nucleus (Figure 4F) also verified the results obtained using live-cell imaging. Moreover, the CCK8 assay suggested that the viability of HL60-type AML cells was significantly reduced upon exposure to 20 μM PTL for 24 h (Figure 4G).

**Figure 5:**
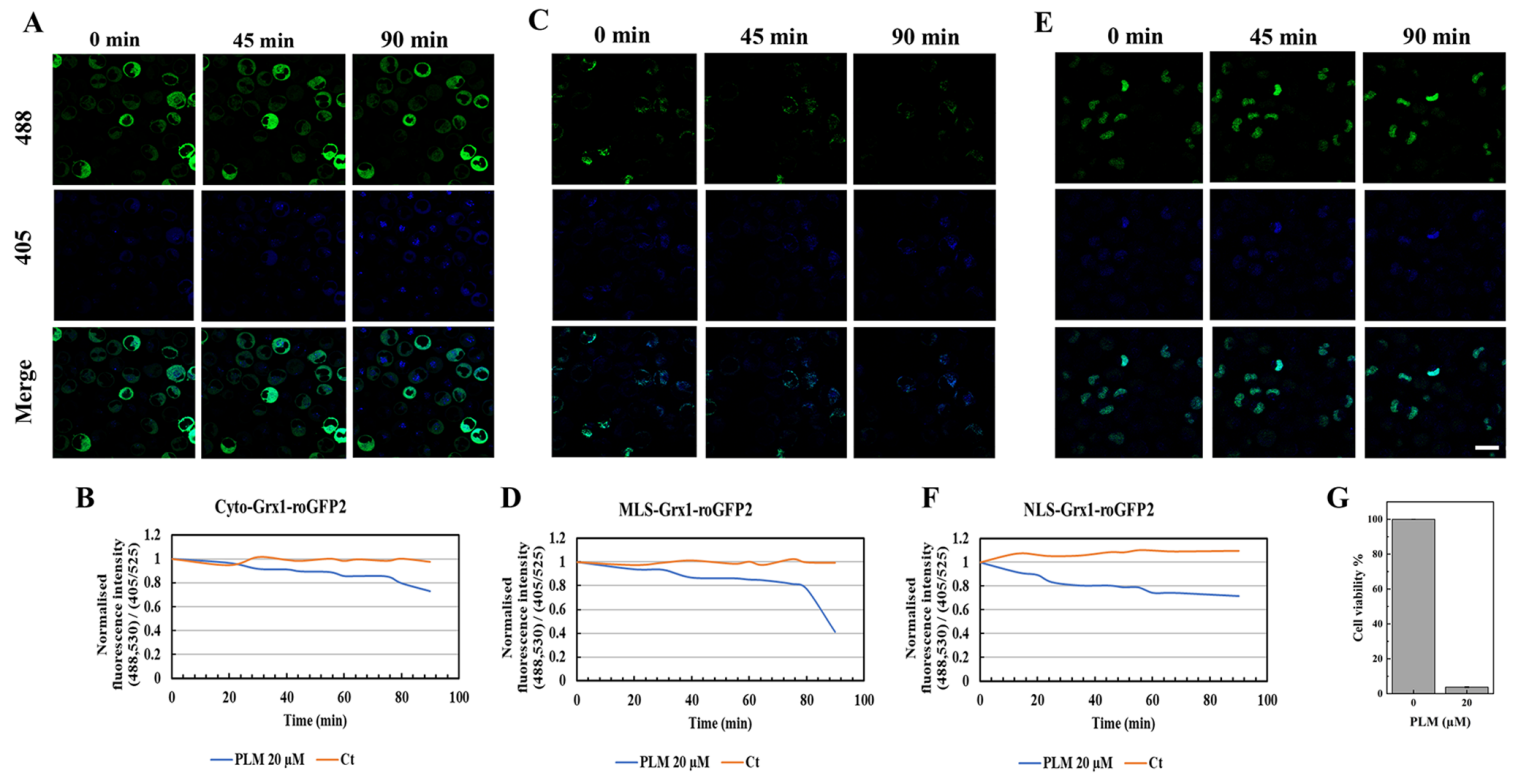
Representative live-cell images of the changes in GSH/GSSG levels in various subcellular compartments (cytosol, mitochondria, and nucleus) of AML cells in response to the glutaminase inhibitor PLM (20 μM). The AML cells expressing the probes Cyto-Grx1-roGFP2, MLS-Grx1-roGFP2, and NLS-Grx1-roGFP2 in the (A) cytosol, (C) mitochondria, and (E) nucleus, respectively, imaged every 5 min for a total of 90 min series after treatment with 20 μM PLM. Quantification of live-cell imaging results showing the normalized fluorescence signal intensity ratio (488,530/405,525) of (B) Cyto-Grx1-roGFP2, (D) MLS-Grx1-roGFP2, and (F) NLS-Grx1-roGFP2 measured using Imaris software and plotted over time in response to PLM. Scale bar: 20 μm. (G) Cell viability of PLM-treated HL60 cells after 24 h, whereas the treatment group is significantly different from the control group. One-way ANOVA was used to compare the differences between different groups. Data are presented as the mean ± SD, *n* ≥ 3.

Likewise, treating the AML stable cell lines expressing Cyto-Grx1-roGFP2 (Figure 5A), MLS-Grx1-roGFP2 (Figure 5C), and NLS-Grx1-roGFP2 (Figure 5E) with 20 μM PLM demonstrated a sharp decrease in GSH/GSSG levels in different subcellular compartments, including the cytosol, mitochondria, and nucleus, conferring the sensitivity of the GSH/GSSG pool in these organelles to PLM. The fluorescence signal intensity in the cytosol (Figure 5B), mitochondria (Figure 5D), and nucleus (Figure 5F) was also comparable to the results of live-cell imaging. Moreover, the CCK8 assay suggested that the viability of HL60-type AML cells was significantly reduced upon exposure to 20 μM PLM for 24 h (Figure 5G).

### Chemotherapeutic Drug Interaction with Enzymes Involved in the GSH Metabolic Pathway

The GSH metabolic pathway showed a varied distribution of the GSH and enzymes involved in its synthesis in different subcellular compartments, such as the cytosol, mitochondria, nucleus, and ER (Figure S4). The five key enzymes (GPX1, GSTP1, GSS, GR, and GLS) involved in GSH biosynthesis were found in specific subcellular compartments.^9^ They maintain the homeostasis of reduced and oxidized GSH levels to combat cell oxidative stress. To further explore the mechanism behind the change in GSH/GSSG levels and investigate how drugs affect the GSH metabolism, as observed in live-cell imaging of different subcellular compartments, molecular docking of enzymes was performed with CB-839, PTL, and PLM. It has been reported that CB-839 disrupts glutamine metabolism and significantly impairs antioxidant GSH synthesis, resulting in increased mitochondrial ROS and apoptotic cell death.^16,38^

**Figure 6:**
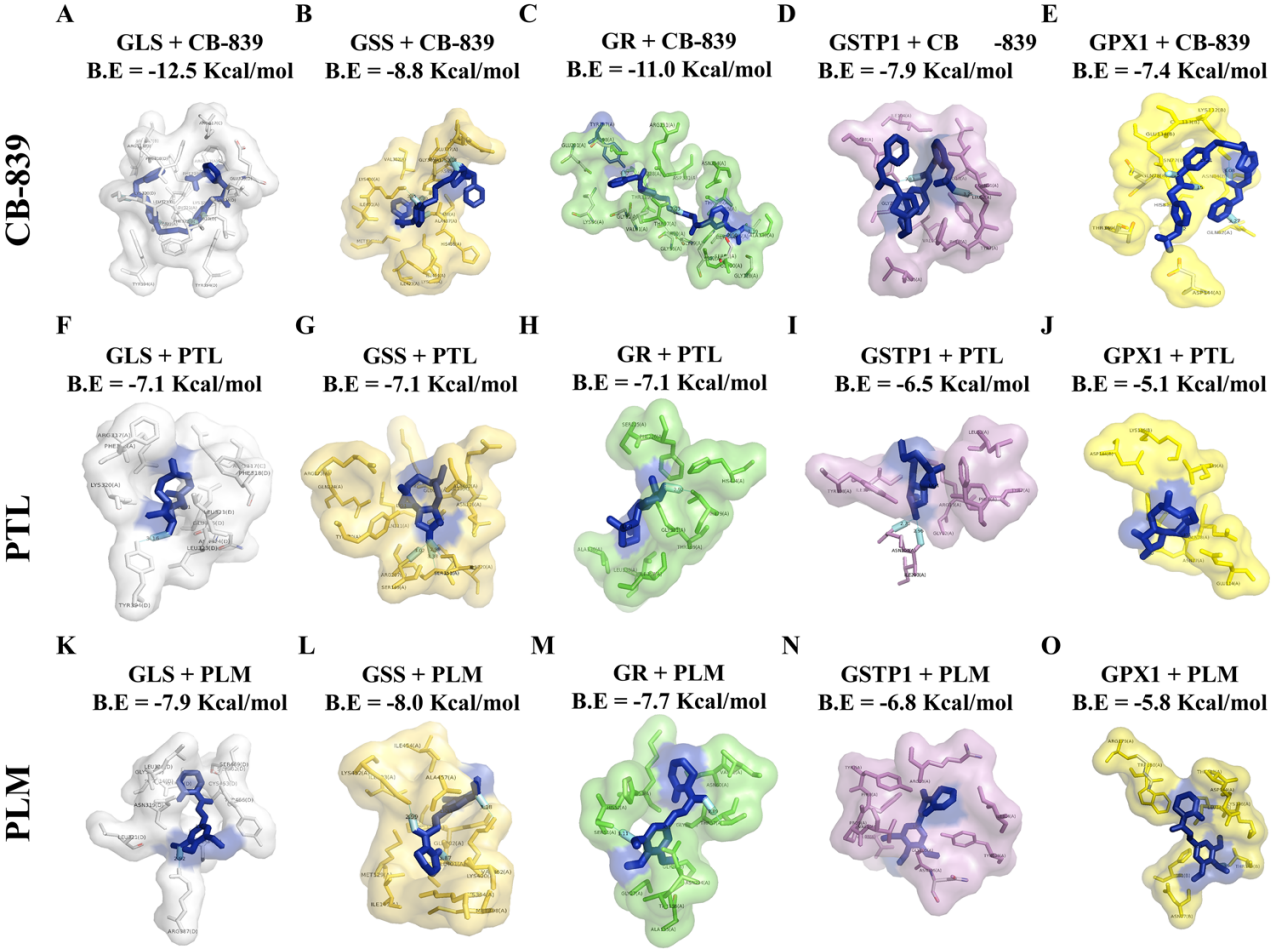
Molecular docking results for the interaction of CB-839, PTL, and PLM with various enzymes involved in the glutathione metabolic pathway. The complex and binding energy of CB-839, PTL, and PLM to GLS, GSS, GR, GSTP1, and GPX1 (A–E, F–J, and K–O), respectively.

**Figure 7:**
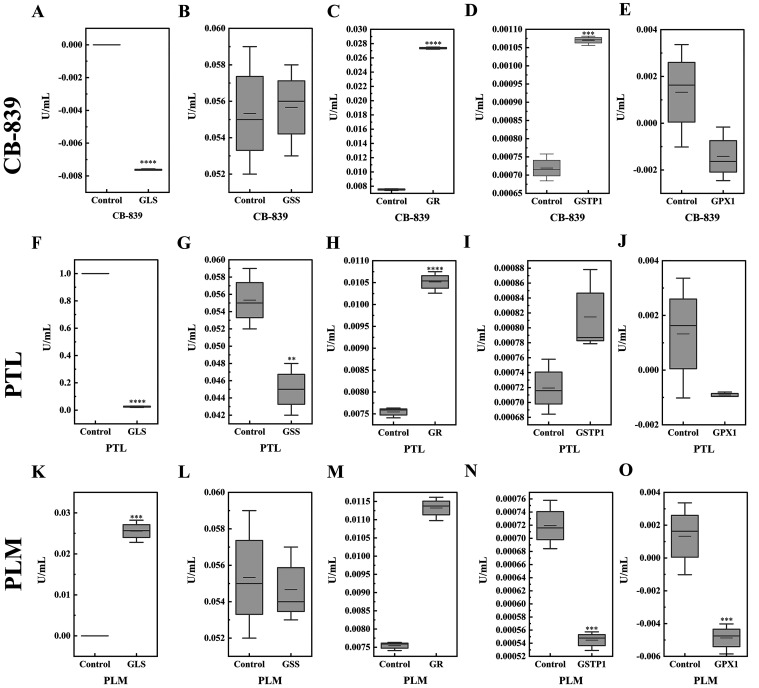
Enzymatic activity assay results for the interaction of CB-839, PTL, and PLM with various enzymes involved in the glutathione metabolic pathway. The enzymatic activity assay results of GLS, GSS, GR, GSTP1, and GPX1 (A–E, F–J, and K–O), respectively, after treatment with 1 μM CB-839, 20 μM PTL, and 20 μM PLM, for 90 min in HL60 AML cells. One-way ANOVA was used to compare the differences between different groups. (****P < 0.0001, ***P < 0.001,**P < 0.01,*P < 0.05). Data are presented as the mean ± SD, *n* ≥ 3.

So, the cells were treated with 1 μM CB-839 (Figure 6A–E), 20 μM PTL (Figure 6F–J), and 20 μM PLM (Figure 6K–O) for 90 min to determine the enzymatic activity in AML cells. The results showed that CB-839, besides its reported target GLS (Figure 6A), had a strong interaction with GPX1 (Figure 6E), as the binding energy of −7.4 Kcal/mol showed a stable complex formation between the enzyme and the drug. Meanwhile, CB-839 had an inhibitory effect on the enzymatic activity of GLS and GPX1 (Figure 7A,E). Besides, PTL had an inhibitory impact on the leukemic GSH/GSSG level and perturbs glutathione homeostasis,^4^ and PLM, a potent and multifunctional anti-AML agent, interfered with cellular GSH metabolism.^39−41^ Similarly, molecular docking results of PTL exhibited a strong interaction and stable complex formation with GLS (Figure 6F) and GSS (Figure 6G), each having a binding energy of −7.1 Kcal/mol, besides its known target GPX1 (Figure 6J). PTL inhibited the activity of GLS and GSS (Figure 7F,G,J). Likewise, the molecular docking results of PLM indicated the compelling interaction and stable complex formation with GPX1 (Figure 6O), showing a binding energy of −5.8 Kcal/mol apart from its previously proclaimed target GSTP1 (Figure 6N), while PLM also showed an inhibitory effect on GPX1 activity (Figure 7N,O).

These results provided evidence of some potential targets in the GSH metabolic pathway. We have summarized the findings demonstrated by the sensor system used in the current study in Table 1. First, all three drugs, CB-839, PTL, and PLM, caused a decline in the GSH/GSSG levels in the cytosol, mitochondria, and nucleus. The most sensitive compartments in response to CB-839 were the mitochondria and nucleus, while PTL treatment indicated that the cytosol along with the nucleus as the most sensitive compartments, and all three compartments were responsive to PLM. Second, it is known that the enzymes GPX1, GSTP1, GSS, GR, and GLS are involved in GSH biosynthesis,^9^ and we discovered the enzymatic activity of GLS and GPX1 was downregulated in the mitochondria and cytosol after CB-839 treatment.

**Table 1:** Overview of Chemotherapeutic Drug (CB-839, PTL, and PLM) Effect on the Subcellular GSH/GSSG Level and Key Enzymes (GPX1, GSTP1, GSS, GR, and GLS) Involved in Glutathione Metabolism

drugs	CB-839	PTL	PLM
subcellular GSH/GSSG level monitoring by the sensor	cytosol ↓	cytosol ↓	cytosol ↓
	mitochondria ↓	mitochondria ↓	mitochondria ↓
	nucleus ↓	nucleus ↓	nucleus ↓
sensitive compartments	mitochondria	cytosol	cytosol
	nucleus	nucleus	mitochondria
			nucleus
molecular docking	GPX1 > GSTP1 > GSS > GR > GLS	GPX1 > GSTP1 > GLS = GR = GSS	GPX1 > GSTP1 > GR > GLS > GSS
enzymatic assay	GPX1 ↓	GPX1 ↓	GPX1 ↓
	GLS ↓	GLS ↓	GSTP1 ↓
		GSS ↓	
reported targets	GLS	GPX1	GSTP
potential targets	GPX1	GLS	GPX1
		GSS	

Similarly, PTL caused an inhibition of GLS and GSS. Third, we extensively investigated the interactions of the drugs and enzymes using molecular docking, and we discovered stable drug–enzyme complexes that, to the best of our knowledge, have not been reported previously, suggesting some new potential therapeutic targets in the GSH metabolic pathway.

## Conclusions

We successfully constructed the genetically encoded fluorescent sensors (Cyto-Grx1-roGFP2, MLS-Grx1-roGFP2, NLS-Grx1-roGFP2, and ELS-Grx1-roGFP2.iL) for the measurement of GSH/GSSG levels in the four subcellular compartments of AML suspension cells. The established sensor system in AML cells enables GSH/GSSG gradient and basal level profiling at the subcellular level, conferring substantial heterogeneity and investigating the drug-sensitive compartments in AML cells via live-cell fluorescence microscopy. With the GSH/GSSG-specific biosensors, we profiled the response spectrum of different subcellular compartments in AML cells under treatment by various chemotherapeutic drugs. Moreover, the live-cell imaging conferred a compartment-specific decrease in GSH/GSSG levels in response to various drugs, CB-839, PTL, and PLM. The mechanism underlying the fluctuations in GSH/GSSG was revealed through an in-depth study of the enzymatic activity influenced by these drugs in the GSH metabolic pathway. We found some potential therapeutic targets in the GSH metabolic pathway of AML cells, as investigated through docking interaction and enzymatic activity assays. Therefore, this study could help elucidate the mechanism of GSH-mediated redox regulation in AML for the development of novel and more efficient AML treatment strategies.

## Supporting Information Available

Additional experimental methods used in this study, all materials, reagents, instruments, and experimental methods used in this study and more information are described in detail in the Supporting Information (PDF)

## References

[ref1] Sillar J. R.; Germon Z. P.; De Iuliis G. N.; Dun M. D. The role of reactive oxygen species in acute myeloid leukaemia. Int. J. Mol. Sci. 2019, 20, 6003 10.3390/ijms20236003. 31795243 PMC6929020

[ref2] Hao X.; Gu H.; Chen C.; Huang D.; Zhao Y.; Xie L.; Zou Y.; Shu H. S.; Zhang Y.; He X.; et al. Metabolic imaging reveals a unique preference of symmetric cell division and homing of leukemia-initiating cells in an endosteal niche. Cell Metab. 2019, 29, 950–965. 10.1016/j.cmet.2018.11.013. 30581117

[ref3] Rasool M.; Farooq S.; Malik A.; Shaukat A.; Manan A.; Asif M.; Sani S.; Qazi M. H.; Kamal M. A.; Iqbal Z.; Hussain A. Assessment of circulating biochemical markers and antioxidative status in acute lymphoblastic leukemia (ALL) and acute myeloid leukemia (AML) patients. Saudi J. Biol. Sci. 2015, 22, 106–111. 10.1016/j.sjbs.2014.09.002. 25561892 PMC4281600

[ref4] Pei S.; Minhajuddin M.; Callahan K. P.; Balys M.; Ashton J. M.; Neering S. J.; Lagadinou E. D.; Corbett C.; Ye H.; Liesveld J. L.; et al. Targeting aberrant glutathione metabolism to eradicate human acute myelogenous leukemia cells. J. Biol. Chem. 2013, 288, 33542–33558. 10.1074/jbc.M113.511170. 24089526 PMC3837103

[ref5] Zitka O.; Skalickova S.; Gumulec J.; Masarik M.; Adam V.; Hubalek J.; Trnkova L.; Kruseova J.; Eckschlager T.; Kizek R. Redox status expressed as GSH: GSSG ratio as a marker for oxidative stress in paediatric tumour patients. Oncol. Lett. 2012, 4, 1247–1253. 10.3892/ol.2012.931. 23205122 PMC3506742

[ref6] Wu G.; Fang Y.-Z.; Yang S.; Lupton J. R.; Turner N. D. Glutathione metabolism and its implications for health. J. Nutr. 2004, 134, 489–492. 10.1093/jn/134.3.489. 14988435

[ref7] Meister A. Biosynthesis and functions of glutathione, an essential biofactor. J. Nutr. Sci. Vitaminol. 1992, 38, 1–6. 10.3177/jnsv.38.Special_1. 1297717

[ref8] Kalinina E.; Feng J.; Novichkova M.; Nurmuradov N.; Alsaidi A.; Chernov N. N. Glutathione and redox-dependent regulation under the cancer cell drug resistance to cisplatin. Free Radical Biol. Med. 2021, 177, S112 10.1016/j.freeradbiomed.2021.08.172.

[ref9] Forman H. J.; Zhang H.; Rinna A. Glutathione: overview of its protective roles, measurement, and biosynthesis. Mol. Aspects Med. 2009, 30, 1–12. 10.1016/j.mam.2008.08.006. 18796312 PMC2696075

[ref10] Diaz-Vivancos P.; de Simone A.; Kiddle G.; Foyer C. H. Glutathione–linking cell proliferation to oxidative stress. Free Radicals Biol. Med. 2015, 89, 1154–1164. 10.1016/j.freeradbiomed.2015.09.023. 26546102

[ref11] Oestreicher J.; Morgan B. Glutathione: Subcellular distribution and membrane transport. Biochem. Cell Biol. 2019, 97, 270–289. 10.1139/bcb-2018-0189. 30427707

[ref12] Fernandez-Checa J. C.; Kaplowitz N.; Garcia-Ruiz C.; Colell A.; Miranda M.; MARi M.; Ardite E.; Morales A. GSH transport in mitochondria: defense against TNF-induced oxidative stress and alcohol-induced defect. Am. J. Physiol. Gastrointest. Liver Physiol. 1997, 273, G7–G17. 10.1152/ajpgi.1997.273.1.G7. 9252504

[ref13] Rellán-Álvarez R.; Hernández L. E.; Abadía J.; Álvarez-Fernández A. Direct and simultaneous determination of reduced and oxidized glutathione and homoglutathione by liquid chromatography–electrospray/mass spectrometry in plant tissue extracts. Anal. Biochem. 2006, 356, 254–264. 10.1016/j.ab.2006.05.032. 16828049

[ref14] Ahmad M.; Anjum N. A.; Asif A.; Ahmad A. Real-time monitoring of glutathione in living cells using genetically encoded FRET-based ratiometric nanosensor. Sci. Rep. 2020, 10, 992 10.1038/s41598-020-57654-y. 31969596 PMC6976633

[ref15] Owen J. B.; Butterfield D. A. Measurement of Oxidized/Reduced Glutathione Ratio. In Protein Misfolding and Cellular Stress in Disease and Aging, Springer, 2010; pp 269–277.

[ref16] Gregory M. A.; Nemkov T.; Park H. J.; Zaberezhnyy V.; Gehrke S.; Adane B.; Jordan C. T.; Hansen K. C.; D’Alessandro A.; DeGregori J. Targeting glutamine metabolism and redox state for leukemia therapy. Clin. Cancer Res. 2019, 25, 4079–4090. 10.1158/1078-0432.CCR-18-3223. 30940653 PMC6642698

[ref17] Rushworth S. A.; Bowles K. M.; MacEwan D. J. High basal nuclear levels of Nrf2 in acute myeloid leukemia reduces sensitivity to proteasome inhibitors. Cancer Res. 2011, 71, 1999–2009. 10.1158/0008-5472.CAN-10-3018. 21212410

[ref18] Zhang H.; Wang C.; Wang K.; Xuan X.; Lv Q.; Jiang K. Ultrasensitive fluorescent ratio imaging probe for the detection of glutathione ultratrace change in mitochondria of cancer cells. Biosens. Bioelectron. 2016, 85, 96–102. 10.1016/j.bios.2016.04.097. 27156018

[ref19] Zhao N.; Qi J.; Zeng Z.; Parekh P.; Chang C.-C.; Tung C.-H.; Zu Y. Transfecting the hard-to-transfect lymphoma/leukemia cells using a simple cationic polymer nanocomplex. J. Controlled Release 2012, 159, 104–110. 10.1016/j.jconrel.2012.01.007. PMC3322282 22269663

[ref20] Basiouni S.; Fuhrmann H.; Schumann J. High-efficiency transfection of suspension cell lines. Biotechniques 2012, 53, 1–4. 10.2144/000113914. 26307260

[ref21] Cui M.; Abbas G.; Wang D.; Liu Q.; Gong R.; Li M.; Zhang X.-E. Genetically encoded redox biosensor system for H2O2 measurement in four subcellular compartments in acute myeloid leukemia (AML) cells. Sci. China: Life Sci. 2022, 65, 1259–1262. 10.1007/s11427-021-2049-6. 35192125

[ref22] Gutscher M.; Pauleau A.-L.; Marty L.; Brach T.; Wabnitz G. H.; Samstag Y.; Meyer A. J.; Dick T. P. Real-time imaging of the intracellular glutathione redox potential. Nat. Methods 2008, 5, 553–559. 10.1038/nmeth.1212. 18469822

[ref23] Aller I.; Rouhier N.; Meyer A. J. Development of roGFP2-derived redox probes for measurement of the glutathione redox potential in the cytosol of severely glutathione-deficient rml1 seedlings. Front. Plant Sci. 2013, 4, 506 10.3389/fpls.2013.00506. 24379821 PMC3863748

[ref24] Meyer A. J.; Brach T.; Marty L.; Kreye S.; Rouhier N.; Jacquot J. P.; Hell R. Redox-sensitive GFP in Arabidopsis thaliana is a quantitative biosensor for the redox potential of the cellular glutathione redox buffer. Plant J. 2007, 52, 973–986. 10.1111/j.1365-313X.2007.03280.x. 17892447

[ref25] Bhaskar A.; Munshi M.; Khan S. Z.; Fatima S.; Arya R.; Jameel S.; Singh A. Measuring glutathione redox potential of HIV-1-infected macrophages. J. Biol. Chem. 2015, 290, 1020–1038. 10.1074/jbc.M114.588913. 25406321 PMC4294471

[ref26] Zhou H.; Tang J.; Lv L.; Sun N.; Zhang J.; Chen B.; Mao J.; Zhang W.; Zhang J.; Zhou J. Intracellular endogenous glutathione detection and imaging by a simple and sensitive spectroscopic off–on probe. Analyst 2018, 143, 2390–2396. 10.1039/C8AN00102B. 29696271

[ref27] Appenzeller-Herzog C. Glutathione- and non-glutathione-based oxidant control in the endoplasmic reticulum. J. Cell Sci. 2011, 124, 847–855. 10.1242/jcs.080895. 21378306

[ref28] M Green R.; Graham M.; R O’Donovan M.; Chipman J. K.; J Hodges N. Subcellular compartmentalization of glutathione: Correlations with parameters of oxidative stress related to genotoxicity. Mutagenesis 2006, 21, 383–390. 10.1093/mutage/gel043. 17012304

[ref29] Franco R.; Cidlowski J. A. Glutathione efflux and cell death. Antioxid. Redox Signal 2012, 17, 1694–1713. 10.1089/ars.2012.4553. 22656858 PMC3474185

[ref30] Birk J.; Meyer M.; Aller I.; Hansen H. G.; Odermatt A.; Dick T. P.; Meyer A. J.; AppenzellELS-Herzog C. Endoplasmic reticulum: reduced and oxidized glutathione revisited. J. Cell Sci. 2013, 126, 1604–1617. 10.1242/jcs.117218. 23424194

[ref31] Mailloux R. J.; Treberg J. R. Protein S-glutathionlyation links energy metabolism to redox signaling in mitochondria. Redox Biol. 2016, 8, 110–118. 10.1016/j.redox.2015.12.010. 26773874 PMC4731959

[ref32] Kojer K.; Bien M.; Gangel H.; Morgan B.; Dick T. P.; Riemer J. Glutathione redox potential in the mitochondrial intermembrane space is linked to the cytosol and impacts the Mia40 redox state. EMBO J. 2012, 31, 3169–3182. 10.1038/emboj.2012.165. 22705944 PMC3400016

[ref33] Montero D.; Tachibana C.; Winther J. R.; AppenzellELS-Herzog C. Intracellular glutathione pools are heterogeneously concentrated. Redox Biol. 2013, 1, 508–513. 10.1016/j.redox.2013.10.005. 24251119 PMC3830055

[ref34] Bass R.; Ruddock L. W.; Klappa P.; Freedman R. B. A major fraction of endoplasmic reticulum-located glutathione is present as mixed disulfides with protein. J. Biol. Chem. 2004, 279, 5257–5262. 10.1074/jbc.M304951200. 14630926

[ref35] Morgan B.; Sobotta M. C.; Dick T. P. Measuring E(GSH) and H2O2 with roGFP2-based redox probes. Free Radic. Biol. Med. 2011, 51, 1943–1951. 10.1016/j.freeradbiomed.2011.08.035. 21964034

[ref36] Shen B.-y.; Chen C.; Xu Y.-f.; Shen J.-j.; Guo H.-m.; Li H.-f.; Li X.-n.; Kang D.; Shao Y.-h.; Zhu Z.-p.; Yin X.-x.; Xie L.; Wang G.-j.; Liang Y. Is the combinational administration of doxorubicin and glutathione a reasonable proposal?. Acta Pharmacol. Sin. 2019, 40, 699–709. 10.1038/s41401-018-0158-8. 30218071 PMC6786300

[ref37] Al-Abbas N. S.; Shaer N. A. Combination of coumarin and doxorubicin induces drug-resistant acute myeloid leukemia cell death. Heliyon 2021, 7, e06255 10.1016/j.heliyon.2021.e06255. 33786386 PMC7988287

[ref38] Cai T.; Lorenzi P. L.; Rakheja D.; Pontikos M. A.; Lodi A.; Han L.; Zhang Q.; Ma H.; Rahmani M.; Bhagat T. D.; et al. Gls inhibitor CB-839 modulates cellular metabolism in AML and potently suppresses AML cell growth when combined with 5-azacitidine. Blood 2016, 128, 4064 10.1182/blood.V128.22.4064.4064.

[ref39] Liao Y.; Niu X.; Chen B.; Edwards H.; Xu L.; Xie C.; Lin H.; Polin L.; Taub J. W.; Ge Y.; Qin Z. Synthesis and Antileukemic Activities of Piperlongumine and HDAC Inhibitor Hybrids against Acute Myeloid Leukemia Cells. J. Med. Chem. 2016, 59, 7974–7990. 10.1021/acs.jmedchem.6b00772. 27505848 PMC6878111

[ref40] Raj L.; Ide T.; Gurkar A. U.; Foley M.; Schenone M.; Li X.; Tolliday N. J.; Golub T. R.; Carr S. A.; Shamji A. F.; Stern A. M.; Mandinova A.; Schreiber S. L.; Lee S. W. Selective killing of cancer cells by a small molecule targeting the stress response to ROS. Nature 2011, 475, 231–234. 10.1038/nature10167. 21753854 PMC3316487

[ref41] Xiong X.-x.; Liu J.-m.; Qiu X.-y.; Pan F.; Yu S.-b.; Chen X.-q. Piperlongumine induces apoptotic and autophagic death of the primary myeloid leukemia cells from patients via activation of ROS-p38/JNK pathways. Acta Pharmacol. Sin. 2015, 36, 362–374. 10.1038/aps.2014.141. 25619389 PMC4349924

[ref42] Yu S.; Zhang H.; Zhang S.; Zhong M.; Fan H. Ferrite Nanoparticles-Based Reactive Oxygen Species-Mediated Cancer Therapy. Front. Chem. 2021, 9, 651053 10.3389/fchem.2021.651053. 33987168 PMC8110829

[ref43] Ahamed M.; Alhadlaq H. A.; Khan M. A. M.; Akhtar M. J. Selective killing of cancer cells by iron oxide nanoparticles mediated through reactive oxygen species via p53 pathway. J. Nanopart. Res. 2013, 15, 1225 10.1007/s11051-012-1225-6.

[ref44] Zhang W.; Hu S.; Yin J.-J.; He W.; Lu W.; Ma M.; Gu N.; Zhang Y. Prussian Blue Nanoparticles as Multienzyme Mimetics and Reactive Oxygen Species Scavengers. J. Am. Chem. Soc. 2016, 138, 5860–5865. 10.1021/jacs.5b12070. 26918394

