# Supplementary material for: Construction of Genetically Encoded Biosensors to Monitor Subcellular Compartment-Specific Glutathione Response to Chemotherapeutic Drugs in Acute Myeloid Leukemia Cells
Source: Anal Chem. 2023 Jan 26;95(5):2838–47. doi: 10.1021/acs.analchem.2c04255 (PMC9909732; doi:10.1021/acs.analchem.2c04255)
Supplement: Supplementary file 1 — ac2c04255_si_001.pdf [file ac2c04255_si_001.pdf]

Construction of genetically encoded biosensors to monitor subcellular compartment-specific glutathione response to chemotherapeutic drugs in acute myeloid leukemia cells

Ghulam Abbas [a,c #], Mengmeng Cui [a#*], Dianbing Wang [a], Min Li [a], Xian-En Zhang [a,b,c*]

a National Laboratory of Biomacromolecules, Institute of Biophysics, Chinese Academy of Sciences, Beijing 100101, China.

b Faculty of Synthetic Biology, Shenzhen Institute of Advanced Technology, Chinese Academy of Sciences, Shenzhen 518055, China.

c University of Chinese Academy of Sciences, Beijing 100049, China.

#These authors contributed equally to this work.

*Corresponding Author, Email: zhangxe@ibp.ac.cn; cuimengmeng@ibp.ac.cn

Content	Page No.
Supplementary Experimental Section	S-3
Additional Results	S-7
Figure S1	S-7
Figure S2	S-7
Figure S3	S-9
Figure S4	S-10
References	S-11

Supplementary Experimental Section

Chemical reagents

α-lipoic acid (ALA) and N-Methylmaleimide (NMM) were acquired from Sigma-Aldrich (USA). Piperlongumine (PLM) and parthenolide (PTL) were purchased from APExBIO (USA), while telaglenastat (CB-839) was acquired from MedChemExpress (USA). H_2O_2 (30% aqueous stock solution) was from SINOPHARM (Beijing, China). Cytarabine (Ara-C), the glutathione S-transferase (GSTP1) Activity Assay Kit-BC0355, glutaminase (GLS) activity assay kit-BC1455, and puromycin were obtained from Solarbio (Beijing, China). Cisplatin was purchased from AbMole (USA). Doxorubicin (DOX), Glutathione reductase (GR) assay kit-S0055, glutathione peroxidase (GPX1) detection kit-S0056, and cell counting kit-8 (CCK-8) were procured from Beyotime (Shanghai, China). The human glutathione synthetase (GSS) quantitative detection kit was purchased from Huixin Biotech., China. The Fe_3O_4 nanoparticles (Fe_3O_4 NPs) and Prussian blue nanoparticles (PBNPs) were kindly provided by Prof. Yu Zhang of the State Key Lab of Bioelectronics, Southeast University (Nanjing, China) [1, 2].

Cell culture

The human leukemia cell line HL60 and human embryonic kidney (HEK) 293T cells were cultured and maintained in RPMI 1640 medium (Gibco, USA) and DMEM (Gibco, USA), respectively, supplemented with 10% fetal bovine serum (FBS, Gibco, USA)) and 1% penicillin-streptomycin (PS, Life Technologies). All the cells were cultured in humidified air containing 5% CO_2 at 37 °C. 293T cells were kindly gifted by Dr. Guang-Xia Gao (Institute of Biophysics, Chinese Academy of Sciences), while the HL60 cell line was obtained from Huiying-Bio (Shanghai, China).

Sensor plasmids cloning

The primers for PCR amplification, subcellular-targeting sequences, and sensor encoding sequences of Grx1-roGFP2 and Grx1-roGFP2.iL were synthesized by Sangon Biotechnology (Shanghai, China). All enzymes used for the molecular cloning of the biosensors were procured from NEB (NEB, USA). The N-terminus of the sensor encoding sequence of Grx1-roGFP2 was cloned with different subcellular targeting sequences, viz., nuclear export sequence (NES; NSNELALKLAGLDINK), nuclear localization sequence (NLS; PKKKRKVGSSPKKKRKVE), mitochondrial localization sequence (MLS; MSVLTPLLLRGLTGSARRLPVPRAKIHSL), to generate the biosensor for cytosolic (Cyto-Grx1-roGFP2), nuclear (NLS-Grx1-roGFP2), and

mitochondrial (MLS-Grx1-roGFP2) expression, respectively. The N-terminus of the Grx1-roGFP2.iL encoding sequence was fused with the ER localization sequence similar to that of the pCMV/myc/ER plasmid and at the C-terminal ER retention signal (KDEL) for expression in ER. The designed plasmids were subcloned into the pLVX lentiviral vector backbone for expression in mammalian cells. All the constructs were confirmed via DNA sequencing.

Live-cell imaging of subcellular compartments

The AML stable cell lines were stained with Hoechst 33342 (Beyotime, Shanghai, China), Mito-Tracker Red (Beyotime, Shanghai, China), or ER-Tracker Red (KeyGen, Nanjing, China) for 20 min at room temperature in the dark to visualize and confirm the intracellular localization of glutathione probes, which were later washed three times. The glutathione probes were illuminated by a 488 nm laser for imaging. We excited the Grx1-roGFP2 and Grx1-roGFP2.iL using a 488 nm laser line, and imaging data of markers were obtained using 405 nm laser excitation for Hoechst 33342 and 561 nm laser line for Mito-Tracker Red and ER-Tracker Red. All 3D-SIM images of AML stable cell lines were acquired using the Delta-Vision OMX V3 imaging system (GE Healthcare) with a 100× 1.4 NA oil-immersion objective (Olympus, UPlanSApo), solid-state multimode lasers (405 nm, 488 nm, and 561 nm), and electron-multiplying charge-coupled device cameras (Evolve 512×512, Photometrics) in conventional mode with serial z-stack sectioning at 250 nm intervals. Immersion oils with a refractive index of 1.518 were used for live-cell imaging of AML cells in a 35 mm glass-bottom dish (BD Biosciences, Franklin Lakes, NJ, USA). The conventional raw data were deconvoluted, and the image stacks were reconstructed using SoftWoRx 6.1.1 (GE Healthcare, USA) to obtain maximum-intensity projections. The reconstructed image datasets were further processed to calculate the Pearson correlation coefficient (PCC) using SoftWoRx 6.1.1.

Characterization of the GSH/GSSG sensor in AML stable cell lines

The Cyto-Grx1-roGFP2 stable cell line was characterized to determine the detection sensitivity of Grx1-roGFP2 to GSH/GSSG levels in AML cells using flow cytometry. The stable cell lines were treated with the typical GSH enhancer alpha-lipoic acid (ALA) and inhibitor N-methylmaleimide (NMM) at concentrations ranging from 0 to 2 mM for 30 and 60 min, respectively. Besides, the HL60 cells were also treated with H_2O_2 at concentrations ranging from 0 to 500 μM for 2 min. Moreover, the AML cells were treated with different concentrations (0-100 μM) of the chemotherapeutic drug cisplatin for 24 h. While the controls were always treated

with the same amount of solvent. The normalized fluorescence intensity response ratio (488/405 nm) was recorded for HL60 cells stably expressing Cyto-Grx1-roGFP2 in the cytosol 30 min after ALA, 60 min after NMM, 2 min after H_2O_2, and 24 h after cisplatin treatment using flow cytometry (≥ 10, 000 cells), whereas 488 nm and 405 nm were used as excitation wavelengths while 530/30 nm and 525/50 nm filter sets for emission, respectively.

Measurement of GSH dynamics using flow cytometry

GSH dynamics were measured in all four stable cell lines after the above-mentioned chemical treatments using the 405 and 488 nm excitation lasers of the FACSAria IIIu flow cytometry, while 525/50 nm and 530/30 nm were used as emission filters. Each histogram was constructed using the data from at least 10,000 events. The flow cytometric data analysis was performed using FlowJo software.

Cell viability and proliferation assay

The CCK-8 assay (Beyotime, Shanghai, China) was used to determine cell viability according to the manufacturer's instructions. Cells were seeded in a 96-well microplate (10^4 cells/well) along with the aforementioned chemical treatments. Subsequently, 10 µL of CCK-8 solution was added to each well and incubated for 2 h at 37 °C in humidified air containing 5% CO_2. The absorbance was measured at 450 nm using a microplate reader (BioTek, USA).

Enzymatic assays and molecular docking

The HL60 cells were seeded onto six-well plates and treated with 1 µM CB-839, 20 µM PTL, or 20 µM PLM, respectively, for 90 min. The cells were later collected, and the enzymatic activity of GLS, GSS, GR, GSTP1, and GPX1 was determined using the appropriate kit for each enzyme following the manufacturer's instructions. On the other hand, the docking was performed by utilizing the AutoDock Vina [3]. Therefore, three ligands, CB-839, PTL, and PLM, were used for the docking with different proteins. In this study, a total of 5 proteins were analyzed for interaction with each aforementioned drug: GLS (5HL1), GSS (2HGS), GR (1XAN), GSTP1 (10GS), and GPX1 (2F8A). The docking complex obtained using MGL Tools and AutoDock Vina was further visualized using the PyMOL visualization system to analyze the bonding and interactions in the complex.

Statistical analysis

Data are presented as the mean ± standard deviation (SD) unless otherwise stated, and all the experiments were independently repeated at least three times. Statistical analyses were

performed using OriginPro and GraphPad Software. One-way analysis of variance (ANOVA) was used to compare differences between different groups (****$P<0.0001$, ***$P<0.001$,**$P<0.01$,*$P<0.05$).

Additional Results

Directed subcellular localization of GSH/GSSG probes in AML cell stable lines

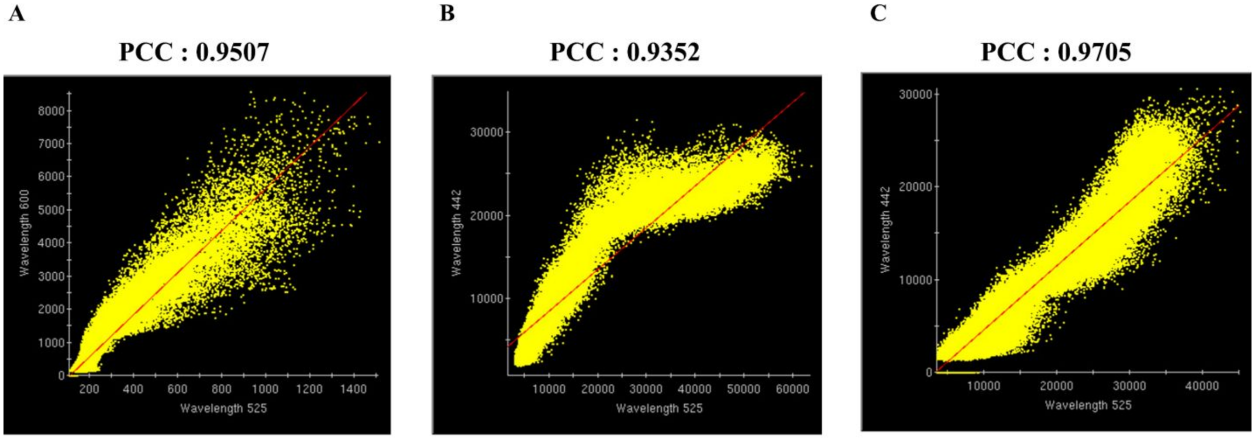

Figure S1. Panels (A, B, and C) represent the Pearson's correlation coefficient (PCC) between the glutathione probe signal and the tracking dye.

Characterization of glutathione sensor in AML cells by live-cell imaging

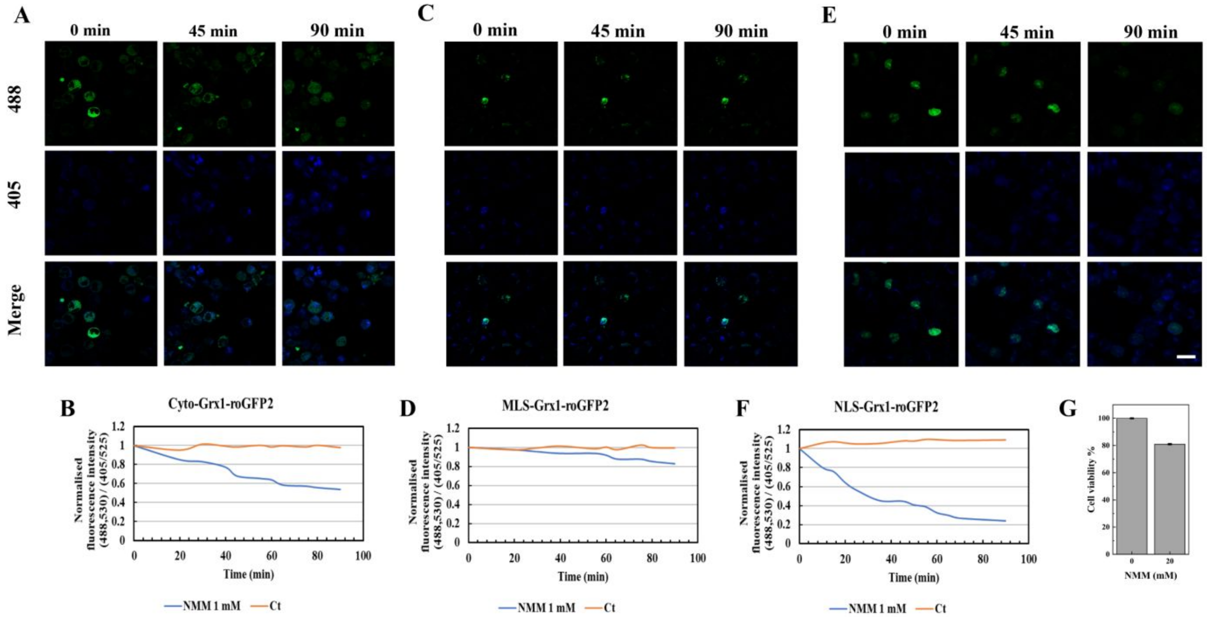

Figure S2. The representative live-cell images of the changes in GSH/GSSG levels in various subcellular compartments (cytosol, mitochondria, and nucleus) of AML cells in response to NMM (1 mM). The AML cells expressing the probes Cyto-Grx1-roGFP2, MLS-Grx1-roGFP2, and NLS-Grx1-roGFP2 in (A) cytosol, (C) mitochondria, and (E) nucleus, respectively, imaged every 5 min for a total of 90 min series after treatment with 1 mM NMM. Quantification of live-cell imaging results showing the normalized fluorescence signal intensity ratio (488,530/405,525) of (B) Cyto-Grx1-roGFP2, (D) MLS-Grx1-roGFP2, and (F) NLS-Grx1-roGFP2 measured using the Imaris software and plotted over time in response to NMM.

Scale bar: 20 μm. (G) Cell viability of NMM treated HL60 cells after 24 h, whereas the treatment group is significantly different from the control group. One-way ANOVA was used to compare the differences between different groups. Data are presented as the mean ± SD, n≥3.

Effect of NPs alone or in combination with Ara-C on GSH/GSSG levels in AML cells.

To study the effect of NPs on AML cells, the Cyto-Grx1-roGFP2-expressing stable cell line was respectively treated with PBNPs and Fe_3O_4 NPs at different concentrations for 24 h. Moreover, these two NPs were added to the Cyto-Grx1-roGFP2-expressing stable cell line combined with 1 μM Ara-C to investigate the collective effect of NPs and chemotherapeutic drugs. This demonstrated that PBNPs caused an increase in the GSH/GSSG levels in AML cells in a concentration-dependent manner (Figure S3A). While Ara-C impaired the GSH/GSSG levels induced by PBNPs (Figure S3B). The response ratio showed that Fe_3O_4 NPs downregulated the GSH/GSSG levels in AML cells in a concentration-dependent manner ranging from 0 to 100 μg/mL (Figure S3C), which displayed a synergistic effect with Ara-C and further decreased the GSH/GSSG levels (Figure S3D).

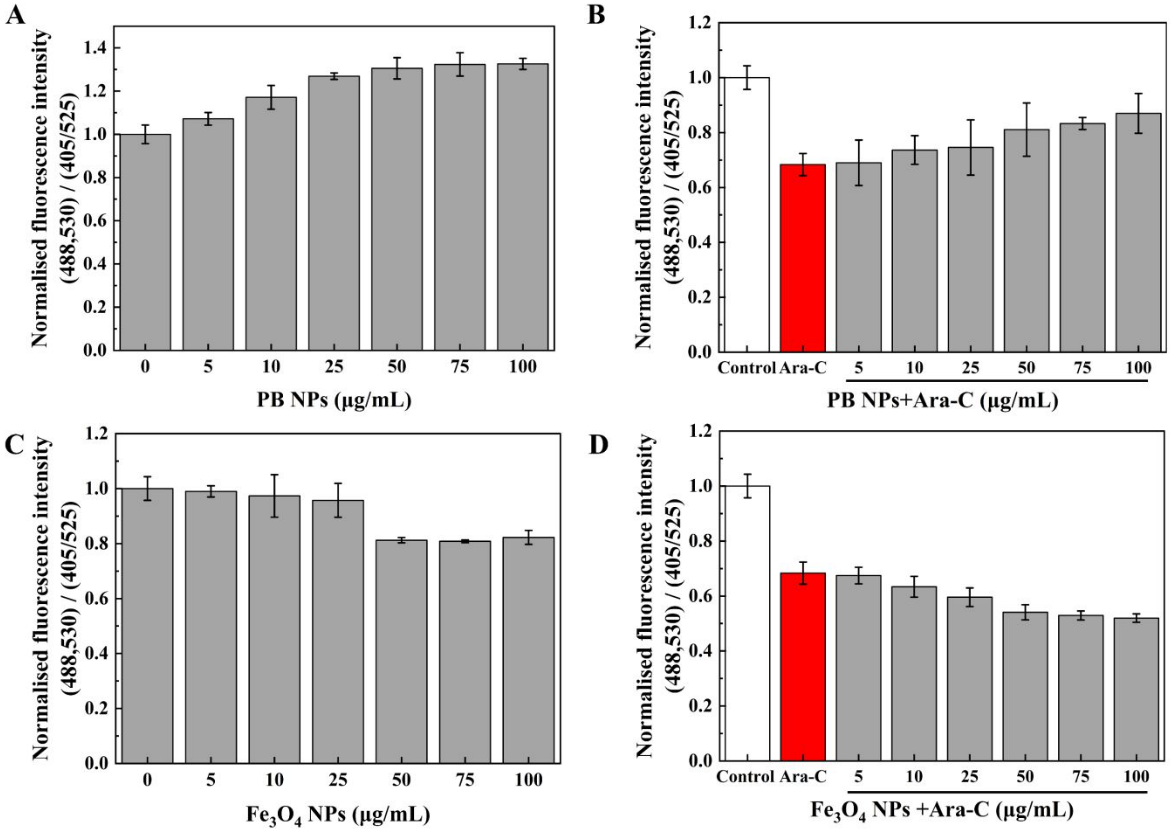

Figure S3. Effect of Prussian blue nanoparticles (PBNPs) and Fe_3O_4 nanoparticles (Fe3O4 NPs) alone or in combination with cytarabine (Ara-C) on GSH/GSSG levels (A, B). The normalized fluorescence signal response ratio (488/405 nm) of Grx1-roGFP2 stably ex-pressed in the cytosol of HL60 cells to (A) PBNPs alone, (B) PBNPs in combination with Ara-C, (C) Fe3O4 NPs alone (D), and Fe_3O_4 NPs in combination with Ara-C after 24 h determined using flow cytometry (≥ 10, 000 cells). Bandpass 530/30 nm and 525/50 nm emission filters were used for 488 nm and 405 nm excitation wavelengths, respectively. One-way ANOVA was used to compare the differences between different groups. Data are presented as the mean ± SD, n≥3.

GSH metabolic pathway

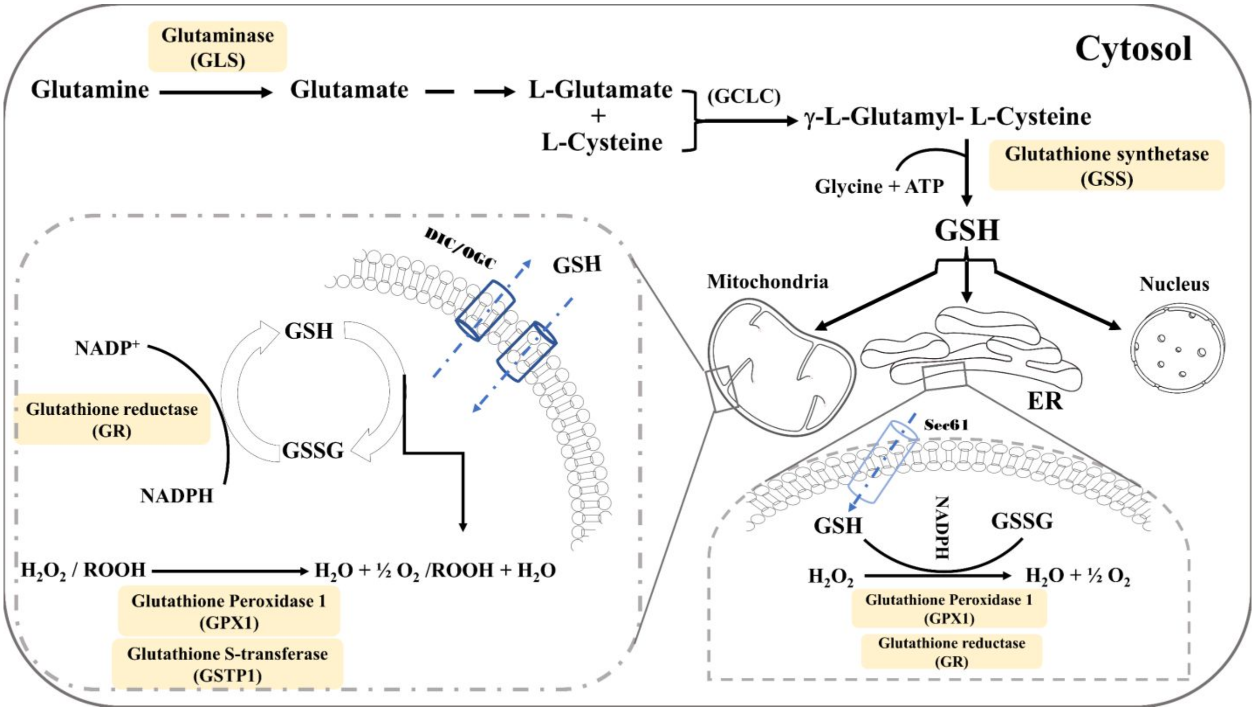

Figure S4. Glutathione metabolic pathway and varied distribution of various enzymes in distinct subcellular compartments.

References

1. Chen, Z. P.; Zhang, Y.; Zhang, S.; Xia, J. G.; Liu, J. W.; Xu, K.; Gu, N., Preparation and characterization of water-soluble monodisperse magnetic iron oxide nanoparticles via surface double-exchange with DMSA. *Colloids and Surfaces A: Physicochemical and Engineering Aspects* **2008,** *316* (1), 210-216.

2. Zhang, W.; Hu, S.; Yin, J.-J.; He, W.; Lu, W.; Ma, M.; Gu, N.; Zhang, Y., Prussian Blue Nanoparticles as Multienzyme Mimetics and Reactive Oxygen Species Scavengers. *Journal of the American Chemical Society* **2016,** *138* (18), 5860-5865.

3. Trott, O.; Olson, A. J. J. J. o. c. c., AutoDock Vina: improving the speed and accuracy of docking with a new scoring function, efficient optimization, and multithreading. **2010,** *31* (2), 455-461.
